# Drivers of caregiver impact in Duchenne muscular dystrophy: a cohort study

**DOI:** 10.1186/s41687-022-00421-6

**Published:** 2022-03-10

**Authors:** Carolyn E. Schwartz, Roland B. Stark, Katrina Borowiec, Bruce D. Rapkin

**Affiliations:** 1 DeltaQuest Foundation, Inc., 31 Mitchell Road, Concord, MA 01742 USA; 2 Departments of Medicine and Orthopaedic Surgery, Tufts University Medical School, Boston, MA USA; 3 Department of Measurement, Evaluation, Statistics, and Assessment, Boston College Lynch School of Education and Human Development, Chestnut Hill, MA USA; 4 Department of Epidemiology and Population Health, Albert Einstein College of Medicine, Bronx, NY USA

**Keywords:** Duchenne muscular dystrophy, Caregiver impact, Demographic, Quality of life, Stress, Behavioral, Cognitive, Resilience, COVID

## Abstract

**Background:**

In our companion paper, we addressed the interplay between caregiver impact, out-of-pocket expenditures, and Duchenne Muscular Dystrophy (DMD) disability. We found that DMD caregiver impact could be characterized by four Latent Profile Analysis impact profiles: lowest, lower middle, upper middle, and highest impact. The impact on caregivers was often but not always worse with greater out-of-pocket expenditures. Further, while the lowest-, lower-middle, and highest-impact profiles reflected low, moderate and high disability-related caregiver burden, respectively, the upper-middle profile group was quite variable in level of disability across domains. To better understand the four caregiver-impact profiles, we examine how a comprehensive set of psychosocial factors differentiate the four profile groups.

**Methods:**

Psychosocial factors assessed included demographic characteristics, quality of life (QOL), stress, cognitive appraisal, reserve-building, and general and COVID-specific resilience. Linear modeling examined relationships between impact profiles and psychosocial factors. We used effect size rather than p-value as the criterion for determining relevance of the broad range of characteristics examined.

**Results:**

Multivariate analyses implicated stress and environmental mastery, appraisal sampling of experience, COVID-specific variables, appraisal standards of comparison, appraisal goals, demographics, appraisal combinatory algorithm, reserve-building, and resilience, in order of prominence (average eta^2^ = 0.29, 0.29, 0.16, 0.15, 0.09, 0.07, 0.07, 0.06, 0.05, and 0.02, respectively). On the whole, comparisons of highest-versus-lowest impact profiles revealed more and larger differences than comparisons of upper-middle versus lower-middle impact profiles. Life stress, goals, and reserve-building activities had a smaller differentiating effect in the middle groups.

**Conclusion:**

A more comprehensive ‘story’ about DMD caregiver impact involves life stress, environmental mastery, COVID-specific variables, and cognitive and behavioral factors. Implications are discussed for coaching interventions to support DMD caregivers.

## Introduction

A substantial body of research has documented the broad range of demographic and psychosocial factors associated with better and worse health outcomes among caregivers. In addition to sociodemographic characteristics such as education, employment status, and racial differences in health outcomes [1], other determinants of worse outcomes cited include stress [2–4], anxiety severity [5], health behaviors, and being overweight [6, 7].

Cognitive and behavioral factors, however, can also be protective against adverse effects of challenging life circumstances. Research on cognitive appraisal processes indicates that how an individual thinks about quality of life (QOL) can mediate and/or moderate the success of clinical and/or surgical interventions [8, 9], can enable or impair one’s ability to adapt to challenging life circumstances [10, 11], and can be associated with differences in sense of control [12] and optimism [13]. Behavioral factors that are particularly relevant are active, stimulating pursuits known as *reserve-building activities* [14]. Specifically, physical exercise, reading, creative hobbies, and spiritual practice may promote better health outcomes across the health-illness spectrum [14–16] and over the course of disease progression [17, 18]. Reserve theory posits that such activities promote resilience by stimulating multiple parts of the brain, enabling it to remain flexible and with higher plasticity [14, 19–22], and facilitating more adaptive ways of coping [23, 24].

The present study builds on our companion paper [25], which found that Duchenne Muscular Dystrophy (DMD) caregiver impact and a care recipient’s disability domains were meaningfully summarized in four impact profiles generated by Latent Profile Analysis (LPA). To summarize briefly the results of this companion paper, the four profiles reflected average degree of impact on the DMD Caregiver Impact measure (DCI): lowest, lower middle, upper middle, and highest impact. The lowest impact group had DMD care recipients with the best mobility, cognitive, and upper extremity functioning proxy scores, and their worst domain was strength impact. The highest impact group had the worst (i.e., highest) scores on all of the DCI impact subscales. The lower-middle impact group had DMD care recipients with consistent, moderate levels of disability across all domains. The upper-middle impact group had DMD care recipients whose disability was quite variable across domains: poor upper extremity functioning, mobility, and negative affect, but relatively high functioning on cognitive and strength impact domains. Of note, the four profiles did not differ appreciably on caregiver or care recipient positive emotions scores.

In the present work, we hypothesize that factors in addition to than their child’s disability and age drive DMD caregiver group differences. This study thus examined the associations between specific psychosocial factors and impact profiles in a sample of DMD caregivers. The psychosocial factors examined included demographic, QOL, life stress, resilience, COVID-related, reserve-building, and cognitive appraisal processes.

## Methods

### Sample and procedure

To facilitate the readers’ task, we reiterate information provided fully in our companion paper [25]. This study recruited participants via Rare Patient Voice, LLC; patient-advocacy groups; and word of mouth (i.e., snowball technique). Eligible participants were age 18 or older, able to complete an online questionnaire, and were providing caregiving support to a family-member with DMD at least two years old, usually their son. Caregiver-participants with motor, visual, and/or other problems that made it difficult for them to complete the web-based survey instrument enlisted the assistance of a household member to enter their survey answers. This survey was administered through the HIPAA-compliant, secure Alchemer engine (www.alchemer.com) from June to November 2020. Dillman’s Tailored Design Method [26] was followed to maximize response and data quality.

Recruitment was stratified by age of the caregiver’s child with DMD: 2–7, 8–12, 13–17, and ≥ 18. These strata broadly correspond to the disease-related phases of progression: ambulatory (age 2–7), transitional (up to age 12), and non-ambulatory (age ≥ 3), with increasing dependence and involvement of other systems as the person ages into adulthood (age ≥ 18). If caregivers had more than one person with DMD for whom they were providing caregiving support, they were asked to report on the eldest or most disabled person (the index patient). Caregivers were paid $75 honoraria for their time completing the survey. The protocol was reviewed and approved by the New England Independent Review Board (NEIRB #20201623), and all participants provided informed consent before beginning the survey.

### Measures

The following person-reported outcomes (PROs) were used to describe caregiver-impact groups created by LPA [25].

*Demographic characteristics* included year of birth, gender, cohabitation/marital status, employment status, ethnicity, race, education, height, weight, with whom the person lived, and smoking status.

*Quality of Life* was assessed using a battery of brief, standardized tools. The PROMIS-10 General Health is a ten-item measure of physical and mental health [27]. Its items assess core domains of health and functioning, including overall physical health, mental health, social health, pain, fatigue, and overall perceived QOL. The NeuroQOL Positive Affect and Well-Being is a 9-item measure of well-being [28]. It enables the evaluation of positive health processes [29]. The Ryff Environmental Mastery is a 7-item subscale of the Ryff Psychological Well-Being measure that assesses how well the individual feels able to deal with the demands of her/his environment [30].

*Stress* was measured in three ways. First, via the Urban Life Stress Inventory [31, 32], the respondent was asked to indicate how much stress s/he experienced during the past 12 months, across 18 areas of life, using a five-level rating scale (none to extreme). Second, financial strain was measured by asking about difficulty paying bills [33], an item that yields fewer missing values than a question about household income[30] and is a better indicator of financial well-being [34]. Third, an item from the Work Productivity and Activity Impairment measure [35, 36] assessed work hours missed in the past week due to DMD caregiving.

*COVID-related variables* were studied using selected items from the United States National Institutes of Health (NIH) Office of Behavioral and Social Sciences Research and the NIH Disaster Research program [37]. Items queried whether anyone in the household was or had been infected with the novel coronavirus-2019 (COVID). Scale scores reflected continuity of healthcare, social support during the pandemic, and COVID-related isolation, financial hardship, and worry.

*Resilience* was defined as maintaining one’s daily activities despite health challenges. The Centers for Disease Control Healthy Days Core Module [38] was used: two items asked respondents how many of the past 30 days their physical health (Physical Health Problems) or mental health (Mental Health Problems), respectively, was not good. A third item, Activities of Daily Living Impaired (ADL Impaired) asked how many of the past 30 days poor health kept them from doing their usual activities. This score was created using residual modeling in which ADL Impaired (dependent variable) was regressed on Physical Health Problems, Mental Health Problems, and their interaction (predictors). Residuals were saved and multiplied by negative one (− 1). Thus, a high resilience score reflects “over-performance,” or more days than expected that the respondent was able to function despite physical or mental health problems or their synergistic effect.

*Behavioral variables* focused on the nine Current-Reserve-Building Activities subscales from the *DeltaQuest Reserve-Building Measure *[39]: Active in the World (e.g., attending lectures; 3 items), Games (3 items), Outdoors (3 items), Creative (e.g., hobbies involving working with one’s hands; 4 items), Religious/Spiritual (e.g., individual or group religious activities; 3 items), Exercise (4 items), Inner Life (e.g., reading; 3 items), Shopping/Cooking (e.g., cooking as a hobby; 2 items), and Passive Media Consumption (e.g., watching television; 3 items). All but Passive Media Consumption are considered activities that promote neurological reserve [14].

*Cognitive variables* assessed QOL Appraisal, as measured by the QOL Appraisal Profile version 2 Short-Form (QOLAP_v2_ SF) [40]. This 28-item measure assesses the four domains of cognitive-appraisal processes involved when answering QOL measures [41–43]. The Frame of Reference Goal Delineation domain queries what personal goals matter most to one’s QOL [6 items]. Sampling of Experience [4 items] queries recall of, and heuristics for determining experiences relevant to, QOL measures. Standards of Comparison [9 items] queries to whom or to what life stage the individual compares him/herself when thinking about QOL. Combinatory Algorithm [9 items] assesses what aspects of QOL are considered more salient or more important than others. The rating-scale options range from “not at all like me” (1) to “very much like me” (5) or “not applicable/decline” (− 99).

### Statistical analysis

The four impact groups identified in our companion paper [25] via latent profile analysis were used as predictors in analysis-of-variance models (ANOVAs). These ANOVAs tested for group differences on the following dependent variables: demographics, QOL, stress, COVID-specific experience, resilience, reserve-building activities, and QOL appraisal domain. We used effect size (ES) rather than p-value as the criterion for determining relevance. P-values would not be appropriate in cases where groups have been intentionally formed so as to maximize their differences. ES was expressed as explained variance (i.e., eta^2^ for the model) or via Cohen’s *d* for differences between a given two groups [44]. Analyses focused on comparisons between the two groups that differed most on caregiver impact (highest vs. lowest) and between the two intermediate groups (upper-middle vs. lower-middle). The latter comparisons are intended to show the various dimensions on which the two groups with “intermediate”-level impact have meaningful differences.

IBM SPSS version 27 [45] was used for all analyses.

## Results

### Sample

Our companion paper provides descriptive information about the 566 caregivers [25]. Our companion paper [25] and an earlier manuscript [46] from this same study provides descriptive information about the care recipients. Briefly, 44% of the DMD care recipients were ambulatory, 24% were in a transitional phase, and 31% were non-ambulatory. The average number of people with DMD for whom the caregivers are providing support was 1.1 (SD 0.4; range 1 to 5), with 93% of the sample providing support to one child with DMD, 6% to two, and less than one percent to more than two. Ten percent of the caregivers reported that their child was on a breathing machine (C-PAP or Bi-PAP) at night. Table 1 provides descriptive information on the abovementioned PROs. Participants reported scores similar to those of the general population on physical and mental health [47] and positive affect/well-being [48]. Although general-population norms are not published for the Ryff environmental mastery, in our earlier work caregivers reported similar albeit slightly lower scores [46]. Only 2% of the sample endorsed anyone in their household having had COVID.

**Table 1:**
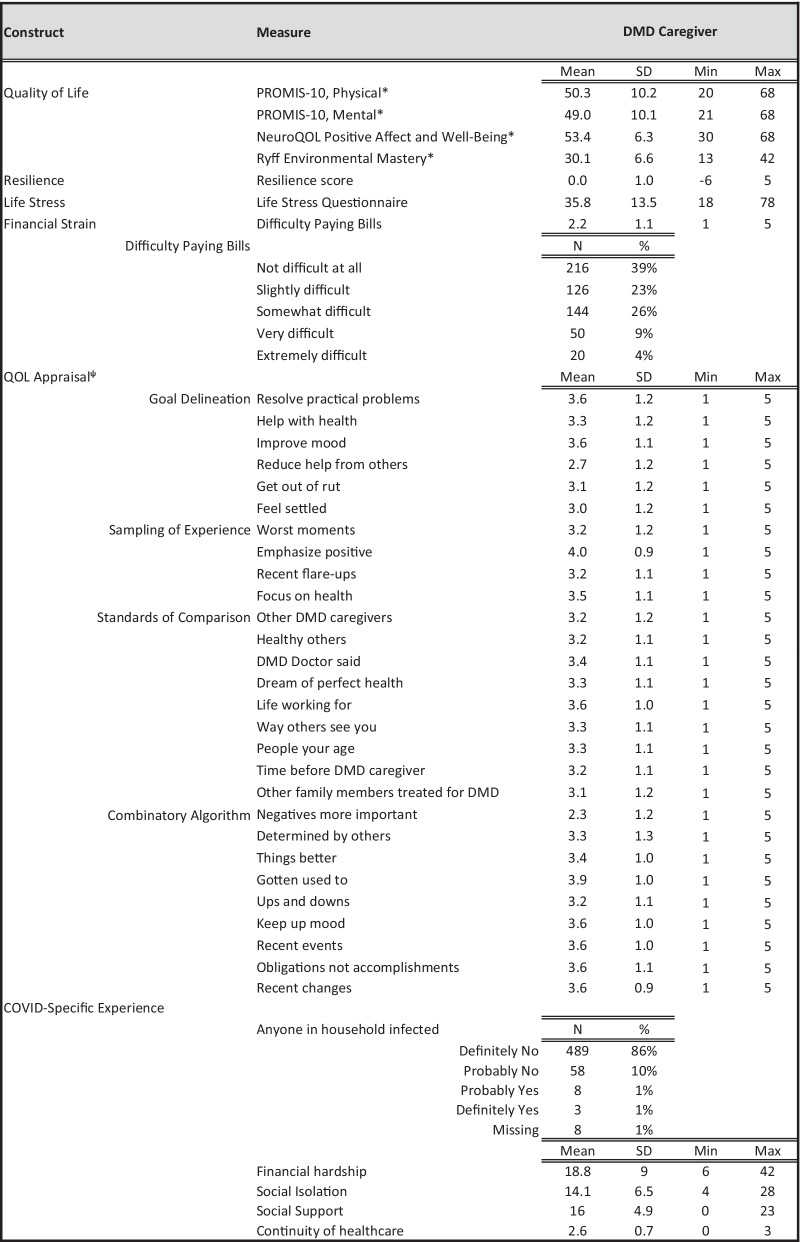
Descriptive statistics of measures used to characterize caregiver-impact groups (N = 566)

* Higher scores indicate better functioning; otherwise higher indicates worse

ψ Higher scores indicate higher endorsement

### Results of ANOVA Models

**Fig. 1:**
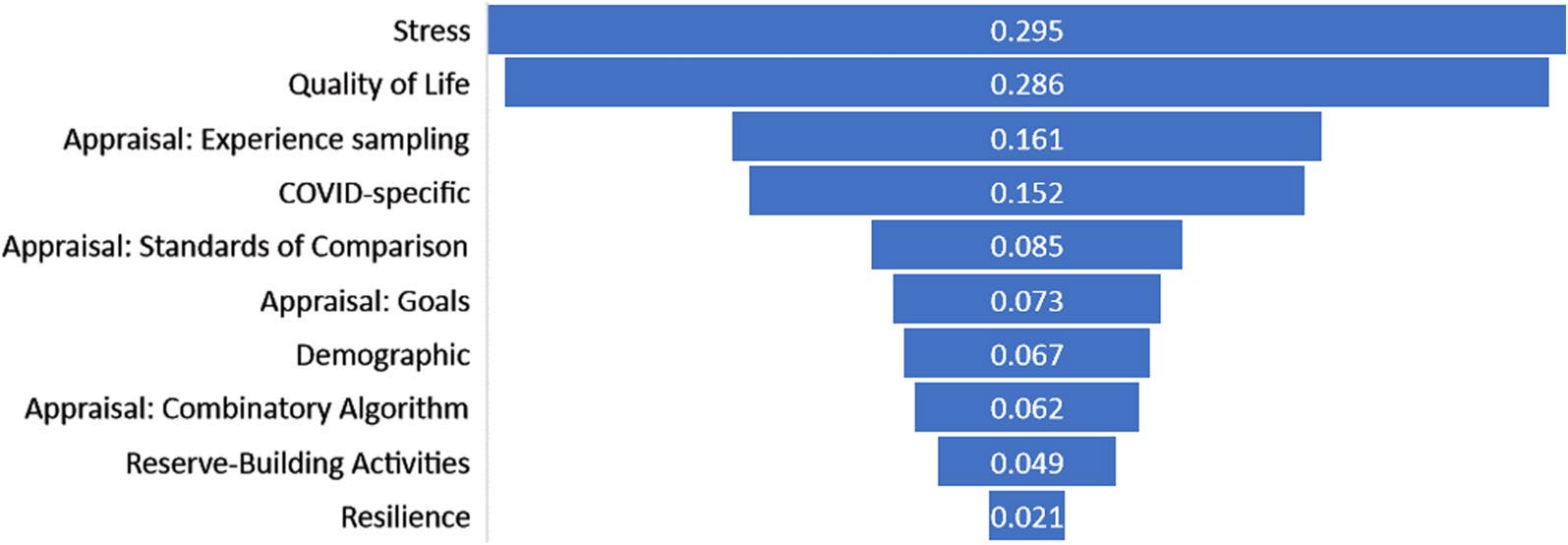
Average explained variance by variable type. The most prominent variable types are shown in this funnel chart in order of average explained variance

Results of ANOVAs examining differences in dependent variables by caregiver group revealed as most prominent, in order of average explained variance: stress and QOL, followed by appraisal sampling of experience, COVID-specific, appraisal standards of comparison, appraisal goals, demographics, appraisal combinatory algorithm, reserve-building, and resilience (average eta^2^ across items = 0.29, 0.29, 0.16, 0.15, 0.09, 0.07, 0.07, 0.06, 0.05, and 0.02, respectively; Fig. 1). Among demographic factors, patient and caregiver comorbidities were most prominent in explaining variance of group membership, followed by caregiver year of birth, body mass index, marital status, ethnicity, gender, recruitment source, whether currently working, and number of DMD care recipients (Table 2). Cohen’s *d* comparing the highest versus lowest-impact groups showed that high-impact groups had more patient and caregiver comorbidities (large ES of d ≥ 0.8), higher body mass index, more DMD care recipients, and younger caregivers (medium ES of d ≥ 0.5). Comparing the upper-middle and lower-middle groups revealed similar direction of effects but generally smaller ES than for the high-versus-low impact groups.

**Table 2:**
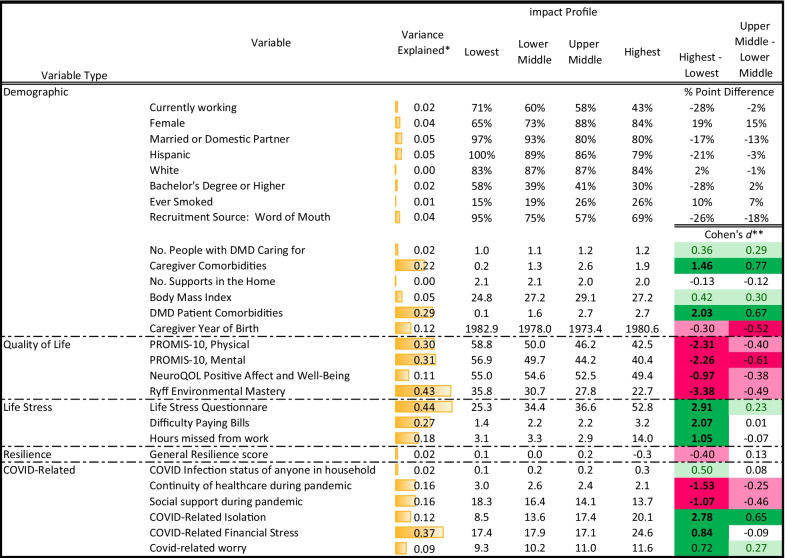
Demographic, quality of life, and stress differences by latent profile group membership

*Variance explained was captured by eta2 for the bivariate relationship

** Conditional formatting indicates the magnitude of effect, with higher saturation reflecting larger ES, and color indicating direction

Results of ANOVAs on QOL variables revealed that the most prominent in distinguishing groups were, in descending order, environmental mastery, mental and physical health, and positive affect/well-being (Table 2). Cohen’s *d* comparing the highest versus lowest-impact groups showed that the former had worse scores on all variables, with large ES for all differences. In comparing the upper-middle and lower-middle groups, the former had worse scores on all, with large ES for mental health and medium ES for the three other QOL scores.

Results of ANOVAs on stress variables revealed that the stressful life events score was most prominent in explaining variance of group membership, followed by difficulty paying bills and then hours missed from work (Table 2). Cohen’s *d* comparing the highest- versus lowest-impact groups showed that high-impact group had worse scores on all variables (large ES). In contrast, the comparison between the upper-middle and lower-middle groups revealed that the upper-middle group had slightly more life stress (small ES) but no differences on difficulty paying bills or work hours missed.

ANOVAs related to COVID-specific variables revealed the greatest profile-group differences for COVID-related financial stress, followed by continuity of healthcare, social support during the pandemic, isolation, and worry. Cohen’s *d* comparing the highest versus lowest-impact groups showed that the high-impact group was more likely to report problems with isolation, COVID-related financial stress, and worry (large ES); and more likely to report COVID infection in their household (small ES). They were also less likely to report having continuity in their DMD care recipient’s healthcare and reported lower social support (large ES). The comparison between the upper-middle and lower-middle groups revealed that caregivers in the upper-middle group tended to report feeling isolated (large ES), and they reported lower social support and continuity of healthcare and more worry (small ES).

ANOVAs revealed that latent-profile group membership explained 2% of the overall variance in resilience. The highest-impact group had somewhat lower resilience than the lowest (small ES; Table 2). There was no difference between the upper-middle and lower-middle groups.

ANOVAs predicting reserve-building variables revealed that profile-group membership’s largest effects were in explaining Cooking/Shopping, Inner Life, Games, Active in the World, and Passive Media Consumption (Table 3). Cohen’s *d* comparing the highest versus lowest-impact groups showed that the highest-impact group reported substantially more Passive Media Consumption (large ES) and Inner Life activities, and less time spent on Cooking/Shopping (both of the latter were medium ES). Along with an inner focus, the highest-impact group counterintuitively reported spending more time on Active in the World, Exercise, Creative, Religious/Spiritual, and Games (small ES). The comparison between the upper-middle and lower-middle groups revealed that the upper-middle group was less likely to engage in Cooking/Shopping and Active-in-the-World activities, and more in Outdoor activities (small ES).

**Table 3:**
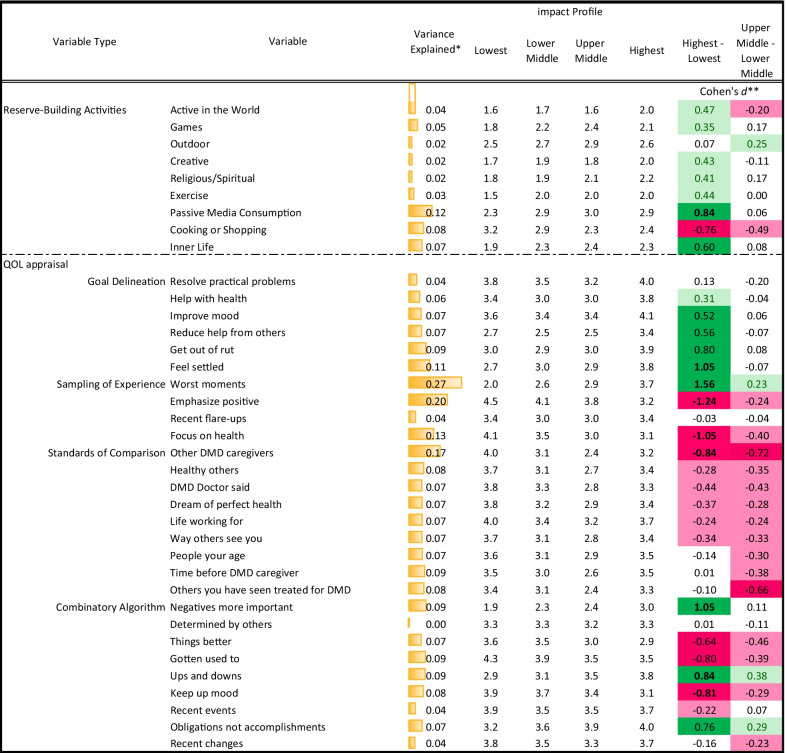
Behavioral and cognitive-appraisal differences by latent profile group membership

*Variance explained was captured by eta2 for the bivariate relationship

** Conditional formatting indicates the magnitude of effect, with higher saturation reflecting larger ES, and color indicating direction

The most prominent differences in QOL appraisal processes explained by group membership were sampling of experience, followed by standards of comparison, goals, and combinatory algorithm (average eta^2^ across items = 0.16, 0.09, 0.07, and 0.06, respectively; Table 3). The high-impact group was notably more likely than the lowest to sample experiences based on recollection of worst moments, and less likely to focus on their health condition or emphasize their positive experiences (large ES). The highest-impact group was less likely to compare themselves to other DMD caregivers (large ES), or to what the DMD doctors said, their dream of perfect health, the way others see them, healthy others, or the life they were working towards (medium ES). Their goals were more related to feeling settled, getting out of a rut, reducing help from others, improving mood (large ES), and improving their own health (small ES). They tended to emphasize the negative, ups and downs, and obligations rather than accomplishments (large ES), and to de-emphasize keeping up their mood, habituation, and things getting better (large ES). Comparisons between the intermediate groups were similar to those above but with smaller ES. There were no differences on goals, but upper-middle-impact caregivers tended to de-emphasize things getting better, habituation, keeping up mood, and recent changes, and to emphasize ups and downs and obligations rather than accomplishments (small ES).

## Discussion

The experience of caregiving for DMD reflects many more variables than a son’s disability and/or age. This work builds on work done by Magliano and colleagues [49].  A more comprehensive story emerges, highlighting differences across the aspects of life examined. Overall, comparisons of highest-versus-lowest impact profiles revealed larger differences than comparisons of upper-middle versus lower-middle impact profiles. Caregivers with the worst impact were younger, had higher body mass index, and reported more comorbidities for themselves and their child with DMD (with other child-disability differences presented in our companion paper [25]). These challenges exacerbate an already difficult situation. This group reported dramatically lower levels of environmental mastery, higher levels of life stress, worse mental and physical health and well-being, worse financial impact, more hours missed from work, and worse COVID-related stress.

Our findings also suggest that cognitive and behavioral processes are relevant to the experience of caregiver impact. Those with the worst impact experience tended to focus their QOL appraisal on more negative ways of sampling experiences and/or patterns of emphasis. These findings are consistent with other research findings in a number of caregiver and patient samples showing that focusing on the negative is associated with worse outcomes [13, 50]. With respect to goals, caregivers with the worst impact profiles tended to center on feeling settled or getting out of a rut, perhaps suggesting that their lives had substantial unpredictability and unpleasurable routines.

The higher-impact caregivers also generally tended to compare themselves less to each of the standards listed. This de-emphasis may reflect a sense of isolation; that their experience is outside the realm of ‘normal’ experience; or even that they do not know others to whom they could compare themselves. Past research has documented that one’s standards of comparison matter in global assessments of QOL. Systematically lower reported QOL has been found to be associated with frequent use of specific standards of comparison: comparing oneself to others without a health condition, and comparing oneself to a time before having a challenging condition [51]. In contrast, better coping has been found to be associated with comparing oneself to others facing similar challenges and/or paying attention to information from a knowledgeable healthcare provider [52].

Caregiver impact was also positively associated with passive-media consumption, a type of activity not shown to build reserve [14] and linked with other problematic health conditions [53–55]. Highest-impact groups also tended to spend more time on solitary, quiet activities (inner life), and less on activities that create hobbies from life chores (cooking/shopping). Unexpectedly, however, this group also reported spending more time on exercise, creative activities, and remaining active and engaged in the world.

While comparisons of the upper-middle versus lower-middle impact profile were similar in direction and magnitude, some differences stood out. More impacted individuals showed distinct tendencies to use common standards of comparison less. Also, the sense of not having an applicable basis for comparison seems particularly germane to these middle groups. The upper-middle group was particularly unlikely to compare themselves to others they have seen treated with DMD, perhaps reflecting a sense of social isolation.

Our findings may be useful for informing potentially multidisciplinary coaching interventions to support DMD caregivers. Such interventions would likely need to respond to a hierarchy of needs, ranging from socioeconomic to psychological. Such interventions might have four foci: (1) helping caregivers develop strategies to manage their child’s fatigue and negative emotions, as these child-disability domains were associated with highest impact; (2) helping caregivers find alternative strategies and/or resources for sharing their responsibilities, such as respite care or services to help with care and home management; (3) helping caregivers find resources that enable a greater sense of control and stability, which might include subsidizing the home accommodations and assistive devices that were found to reduce caregiver impact; and (4) utilizing cognitive-behavioral therapy [56, 57] to facilitate a more positive orientation in the way caregivers sample experiences, to whom they compare themselves, and their patterns of emphasis. This multifocal coaching would be consistent with recent international clinical work using the “What Matters to You?” paradigm [58, 59] to address barriers to health and wellness.

As noted in our companion paper [25], the present study has a number of strengths, including its large sample size, comprehensive measurement of relevant constructs, application of multivariate methods that enable robust inferential statistics, and its reliance on ES rather than p-values to ensure the clinical relevance of findings. Its limitations must, however, be acknowledged. The constructs measured in the Caregiver Impact Measure overlap meaningfully with several of the QOL measures, including physical and mental health and well-being. Thus, the most informative QOL measure is the Ryff Environmental Mastery, which was not coincidentally the most relevant for distinguishing the impact-profile groups. We also did not collect information on type of dystrophin variance the care recipient carried, the child’s education level, whether they received physical therapy, or on their cardiac or respiratory status.

In summary, this study found that in addition to their child’s level of disability, DMD caregivers’ experience of impact varies as a function of their own health and environmental mastery, and of levels of life stress. More impacted caregivers fared worse during the COVID pandemic. Caregivers’ engagement in passive-media consumption was associated with worsened impact, as was a cognitive pattern that focused on the negative. These findings could be useful for creating coaching interventions to help DMD caregivers fare better not only with their child’s DMD trajectory, but also with their own health and well-being.

## Data Availability

The study data are confidential and thus not able to be shared.

## Abbreviations

ADL: Activities of daily living
ANOVA: Analysis of variance
COVID: Novel coronavirus-2019
DMD: Duchenne muscular dystrophy
ES: Effect size
NIH: National Institutes of Health
PRO: Person-reported outcome
PROMIS: Patient-Reported Outcome Measurement Information System
QOL: Quality of life
QOLAP_v2_ SF: QOL Appraisal Profile version 2 Short-Form
